# Application of handmade rubber loop traction assisted defect closure after super minimally invasive surgery of gastric gastrointestinal stromal tumor

**DOI:** 10.1055/a-2589-1585

**Published:** 2025-05-19

**Authors:** Yaoqian Yuan, Kunming Lv, Bo Ning, Qun Shao, Enqiang Linghu, Qianqian Chen

**Affiliations:** 1 651943 Department of Gastroenterology, The First Medical Center of Chinese PLA General Hospital, Beijing, China

The closure of large mucosal defects postendoscopic resection poses a significant challenge. Traditional methods, including clips, through-the-scope clips (TTS clips), and over-the-scope clips (OTS clips), have limitations in terms of cost, complexity, and applicability to large defects 1 2 . While TTS clips are effective for small- to medium-sized defects, they may struggle with larger defects due to their limited opening size and strength. OTS clips, on the other hand, although more robust, require pre-procedure attachment to the endoscope’s tip, which can increase procedural complexity and operative time 3 4 . Additionally, the high cost of OTS clips may limit their widespread use, particularly in resource-limited settings.

We present a case where a handmade rubber loop traction-assisted closure technique was successfully employed to manage a large defect after super minimally invasive surgery (SMIS) of a gastric gastrointestinal stromal tumor (GIST). This innovative approach leverages the elasticity and simplicity of a rubber loop to provide continuous traction, facilitating the approximation of large mucosal defects and enabling secure closure with traditional clips. The technique is cost-effective, easy to implement, and does not require specialized equipment, making it a valuable addition to the endoscopic armamentarium for managing challenging defect closures.

A 32-year-old man was admitted to our hospital with a 3.0 × 2.5-cm gastric submucosal tumor (SMT) in the gastric antrum. We resected the lesion using SMIS to resect the tumor while retaining the mucosa ( Fig. 1 , Video 1 ). The post-SMIS defect is about 4.0 × 5.0 cm. We used a handmade rubber loop to assist recover the mucosal layer to the defect ( Fig. 1 ). We clamped the side of the mucosa and used another clip positioning the normal gastric antrum. When the partially resected mucosal layer largely covered the defect, we proceeded to use traditional clips to close the defect along both sides ( Fig. 1 ). Subsequently, the handmade rubber loop was removed. Finally, the GIST was completely resected, and the defect was completely closed while preserving the original gastric wall tissue.

**Fig. 1:**
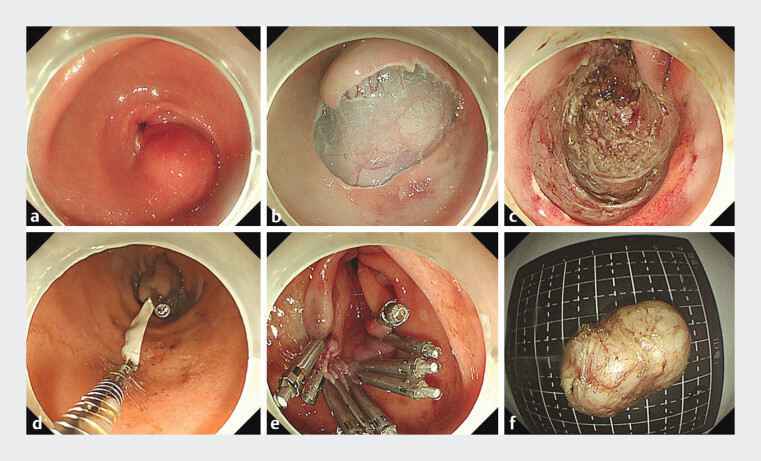
Operation steps of super minimally invasive surgery for the GIST. **a** an SMT was located in the gastric antrum. **b** cutting the mucosal layer to expose the GIST. **c** the GIST was completely removed and a large defect was formed. **d** placing the handmade rubber loop to assist the large defect closure. **e** the defect was completely closed while preserving the original gastric wall tissue. **f** the specimen of the GIST. Abbreviations: GIST, gastrointestinal stromal tumor; SMT, submucosal tumor.

Closure of a large defect in the gastric antrum with the handmade rubber loop after super minimally invasive surgery.Video 1

The handmade rubber loop traction-assisted closure technique offers several advantages, such as simplicity, cost-effectiveness, and efficacy. First, the device is easy to prepare and use, requiring no specialized equipment. Second, the materials used are inexpensive and readily available. Third, the technique effectively approximates large mucosal defects, facilitating secure closure with traditional clips.

Endoscopy_UCTN_Code_TTT_1AO_2AO

## Notes

Endoscopy E-Videos https://eref.thieme.de/e-videos
*E-Videos* is an open access online section of the journal *Endoscopy* , reporting on interesting cases and new techniques in gastroenterological endoscopy. All papers include a high-quality video and are published with a Creative Commons CC-BY license. Endoscopy E-Videos qualify for HINARI discounts and waivers and eligibility is automatically checked during the submission process. We grant 100% waivers to articles whose corresponding authors are based in Group A countries and 50% waivers to those who are based in Group B countries as classified by Research4Life (see: https://www.research4life.org/access/eligibility/ ). This section has its own submission website at https://mc.manuscriptcentral.com/e-videos .

## References

[1] Zhang Q Jin HY Shen ZH. Novel through-the-scope twin clip for the closure of GI wounds: the first experimental survival study in pigs (with videos) Gastrointest Endosc 2021 94 850 858 e2 33965383 10.1016/j.gie.2021.04.027

[2] Sawada A Hirasawa K Sato C Endoscopic Resection with One-Port Placement: A Newly Developed Technique for the Safe Management of Advanced Endoscopic Resection for Gastric Gastrointestinal Stromal Tumors Digestion 2023 104 460 467 37647880 10.1159/000532012 PMC10711755

[3] Ma M Liu S Wang J Closure of a large post-endoscopic submucosal dissection mucosal defect in the duodenum with a novel through-the-scope twin clip Endoscopy 2023 55 E523 E524 36894150 10.1055/a-2024-9901 PMC9998218

[4] Li C Li S Wang T Application of through-the-scope twin clip for defect closure after gastric gastrointestinal stromal tumor transoral super minimally invasive surgery resection Endoscopy 2024 56 E603 E604 10.1055/a-2346-5017 38977030 PMC11281893

